# Supplementary material for: Resolving the full spectrum of human genome variation using Linked-Reads
Source: Genome Res. 2019 Apr;29(4):635–45. doi: 10.1101/gr.234443.118 (PMC6442396; doi:10.1101/gr.234443.118)
Supplement: Supplemental Material [file supp_gr.234443.118_Supplemental_longranger-2.2.zip › longranger-2.2.tar.bz2!/lr2.2-public/lib/go/src/code.google.com/vitess/third_party/py/mock-1.0.1.tar.gz!/mock-1.0.1/html/patch.html]

Patch Decorators — Mock 1.0.1 documentation


### Navigation

- index
- next |
- previous |
- Mock 1.0.1 documentation »

# Patch Decorators¶

The patch decorators are used for patching objects only within the scope of
the function they decorate. They automatically handle the unpatching for you,
even if exceptions are raised. All of these functions can also be used in with
statements or as class decorators.

## patch¶

Note

patch is straightforward to use. The key is to do the patching in the
right namespace. See the section where to patch.

patch(*target*, *new=DEFAULT*, *spec=None*, *create=False*, *spec\_set=None*, *autospec=None*, *new\_callable=None*, *\*\*kwargs*)¶
:   patch acts as a function decorator, class decorator or a context
    manager. Inside the body of the function or with statement, the target
    is patched with a new object. When the function/with statement exits
    the patch is undone.

    If new is omitted, then the target is replaced with a
    MagicMock. If patch is used as a decorator and new is
    omitted, the created mock is passed in as an extra argument to the
    decorated function. If patch is used as a context manager the created
    mock is returned by the context manager.

    target should be a string in the form ‘package.module.ClassName’. The
    target is imported and the specified object replaced with the new
    object, so the target must be importable from the environment you are
    calling patch from. The target is imported when the decorated function
    is executed, not at decoration time.

    The spec and spec\_set keyword arguments are passed to the MagicMock
    if patch is creating one for you.

    In addition you can pass spec=True or spec\_set=True, which causes
    patch to pass in the object being mocked as the spec/spec\_set object.

    new\_callable allows you to specify a different class, or callable object,
    that will be called to create the new object. By default MagicMock is
    used.

    A more powerful form of spec is autospec. If you set autospec=True
    then the mock with be created with a spec from the object being replaced.
    All attributes of the mock will also have the spec of the corresponding
    attribute of the object being replaced. Methods and functions being mocked
    will have their arguments checked and will raise a TypeError if they are
    called with the wrong signature. For mocks
    replacing a class, their return value (the ‘instance’) will have the same
    spec as the class. See the create\_autospec() function and
    *Autospeccing*.

    Instead of autospec=True you can pass autospec=some\_object to use an
    arbitrary object as the spec instead of the one being replaced.

    By default patch will fail to replace attributes that don’t exist. If
    you pass in create=True, and the attribute doesn’t exist, patch will
    create the attribute for you when the patched function is called, and
    delete it again afterwards. This is useful for writing tests against
    attributes that your production code creates at runtime. It is off by by
    default because it can be dangerous. With it switched on you can write
    passing tests against APIs that don’t actually exist!

    Patch can be used as a TestCase class decorator. It works by
    decorating each test method in the class. This reduces the boilerplate
    code when your test methods share a common patchings set. patch finds
    tests by looking for method names that start with patch.TEST\_PREFIX.
    By default this is test, which matches the way unittest finds tests.
    You can specify an alternative prefix by setting patch.TEST\_PREFIX.

    Patch can be used as a context manager, with the with statement. Here the
    patching applies to the indented block after the with statement. If you
    use “as” then the patched object will be bound to the name after the
    “as”; very useful if patch is creating a mock object for you.

    patch takes arbitrary keyword arguments. These will be passed to
    the Mock (or new\_callable) on construction.

    patch.dict(...), patch.multiple(...) and patch.object(...) are
    available for alternate use-cases.

patch as function decorator, creating the mock for you and passing it into
the decorated function:

```
>>> @patch('__main__.SomeClass')
... def function(normal_argument, mock_class):
...     print mock_class is SomeClass
...
>>> function(None)
True
```

Patching a class replaces the class with a MagicMock *instance*. If the
class is instantiated in the code under test then it will be the
return\_value of the mock that will be used.

If the class is instantiated multiple times you could use
side\_effect to return a new mock each time. Alternatively you
can set the return\_value to be anything you want.

To configure return values on methods of *instances* on the patched class
you must do this on the return\_value. For example:

```
>>> class Class(object):
...     def method(self):
...         pass
...
>>> with patch('__main__.Class') as MockClass:
...     instance = MockClass.return_value
...     instance.method.return_value = 'foo'
...     assert Class() is instance
...     assert Class().method() == 'foo'
...
```

If you use spec or spec\_set and patch is replacing a *class*, then the
return value of the created mock will have the same spec.

```
>>> Original = Class
>>> patcher = patch('__main__.Class', spec=True)
>>> MockClass = patcher.start()
>>> instance = MockClass()
>>> assert isinstance(instance, Original)
>>> patcher.stop()
```

The new\_callable argument is useful where you want to use an alternative
class to the default MagicMock for the created mock. For example, if
you wanted a NonCallableMock to be used:

```
>>> thing = object()
>>> with patch('__main__.thing', new_callable=NonCallableMock) as mock_thing:
...     assert thing is mock_thing
...     thing()
...
Traceback (most recent call last):
  ...
TypeError: 'NonCallableMock' object is not callable
```

Another use case might be to replace an object with a StringIO instance:

```
>>> from StringIO import StringIO
>>> def foo():
...     print 'Something'
...
>>> @patch('sys.stdout', new_callable=StringIO)
... def test(mock_stdout):
...     foo()
...     assert mock_stdout.getvalue() == 'Something\n'
...
>>> test()
```

When patch is creating a mock for you, it is common that the first thing
you need to do is to configure the mock. Some of that configuration can be done
in the call to patch. Any arbitrary keywords you pass into the call will be
used to set attributes on the created mock:

```
>>> patcher = patch('__main__.thing', first='one', second='two')
>>> mock_thing = patcher.start()
>>> mock_thing.first
'one'
>>> mock_thing.second
'two'
```

As well as attributes on the created mock attributes, like the
return\_value and side\_effect, of child mocks can
also be configured. These aren’t syntactically valid to pass in directly as
keyword arguments, but a dictionary with these as keys can still be expanded
into a patch call using \*\*:

```
>>> config = {'method.return_value': 3, 'other.side_effect': KeyError}
>>> patcher = patch('__main__.thing', **config)
>>> mock_thing = patcher.start()
>>> mock_thing.method()
3
>>> mock_thing.other()
Traceback (most recent call last):
  ...
KeyError
```

## patch.object¶

patch.object(*target*, *attribute*, *new=DEFAULT*, *spec=None*, *create=False*, *spec\_set=None*, *autospec=None*, *new\_callable=None*, *\*\*kwargs*)¶
:   patch the named member (attribute) on an object (target) with a mock
    object.

    patch.object can be used as a decorator, class decorator or a context
    manager. Arguments new, spec, create, spec\_set, autospec and
    new\_callable have the same meaning as for patch. Like patch,
    patch.object takes arbitrary keyword arguments for configuring the mock
    object it creates.

    When used as a class decorator patch.object honours patch.TEST\_PREFIX
    for choosing which methods to wrap.

You can either call patch.object with three arguments or two arguments. The
three argument form takes the object to be patched, the attribute name and the
object to replace the attribute with.

When calling with the two argument form you omit the replacement object, and a
mock is created for you and passed in as an extra argument to the decorated
function:

```
>>> @patch.object(SomeClass, 'class_method')
... def test(mock_method):
...     SomeClass.class_method(3)
...     mock_method.assert_called_with(3)
...
>>> test()
```

spec, create and the other arguments to patch.object have the same
meaning as they do for patch.

## patch.dict¶

patch.dict(*in\_dict*, *values=()*, *clear=False*, *\*\*kwargs*)¶
:   Patch a dictionary, or dictionary like object, and restore the dictionary
    to its original state after the test.

    in\_dict can be a dictionary or a mapping like container. If it is a
    mapping then it must at least support getting, setting and deleting items
    plus iterating over keys.

    in\_dict can also be a string specifying the name of the dictionary, which
    will then be fetched by importing it.

    values can be a dictionary of values to set in the dictionary. values
    can also be an iterable of (key, value) pairs.

    If clear is True then the dictionary will be cleared before the new
    values are set.

    patch.dict can also be called with arbitrary keyword arguments to set
    values in the dictionary.

    patch.dict can be used as a context manager, decorator or class
    decorator. When used as a class decorator patch.dict honours
    patch.TEST\_PREFIX for choosing which methods to wrap.

patch.dict can be used to add members to a dictionary, or simply let a test
change a dictionary, and ensure the dictionary is restored when the test
ends.

```
>>> from mock import patch
>>> foo = {}
>>> with patch.dict(foo, {'newkey': 'newvalue'}):
...     assert foo == {'newkey': 'newvalue'}
...
>>> assert foo == {}

>>> import os
>>> with patch.dict('os.environ', {'newkey': 'newvalue'}):
...     print os.environ['newkey']
...
newvalue
>>> assert 'newkey' not in os.environ
```

Keywords can be used in the patch.dict call to set values in the dictionary:

```
>>> mymodule = MagicMock()
>>> mymodule.function.return_value = 'fish'
>>> with patch.dict('sys.modules', mymodule=mymodule):
...     import mymodule
...     mymodule.function('some', 'args')
...
'fish'
```

patch.dict can be used with dictionary like objects that aren’t actually
dictionaries. At the very minimum they must support item getting, setting,
deleting and either iteration or membership test. This corresponds to the
magic methods \_\_getitem\_\_, \_\_setitem\_\_, \_\_delitem\_\_ and either
\_\_iter\_\_ or \_\_contains\_\_.

```
>>> class Container(object):
...     def __init__(self):
...         self.values = {}
...     def __getitem__(self, name):
...         return self.values[name]
...     def __setitem__(self, name, value):
...         self.values[name] = value
...     def __delitem__(self, name):
...         del self.values[name]
...     def __iter__(self):
...         return iter(self.values)
...
>>> thing = Container()
>>> thing['one'] = 1
>>> with patch.dict(thing, one=2, two=3):
...     assert thing['one'] == 2
...     assert thing['two'] == 3
...
>>> assert thing['one'] == 1
>>> assert list(thing) == ['one']
```

## patch.multiple¶

patch.multiple(*target*, *spec=None*, *create=False*, *spec\_set=None*, *autospec=None*, *new\_callable=None*, *\*\*kwargs*)¶
:   Perform multiple patches in a single call. It takes the object to be
    patched (either as an object or a string to fetch the object by importing)
    and keyword arguments for the patches:

    ```
    with patch.multiple(settings, FIRST_PATCH='one', SECOND_PATCH='two'):
        ...
    ```

    Use DEFAULT as the value if you want patch.multiple to create
    mocks for you. In this case the created mocks are passed into a decorated
    function by keyword, and a dictionary is returned when patch.multiple is
    used as a context manager.

    patch.multiple can be used as a decorator, class decorator or a context
    manager. The arguments spec, spec\_set, create, autospec and
    new\_callable have the same meaning as for patch. These arguments will
    be applied to *all* patches done by patch.multiple.

    When used as a class decorator patch.multiple honours patch.TEST\_PREFIX
    for choosing which methods to wrap.

If you want patch.multiple to create mocks for you, then you can use
DEFAULT as the value. If you use patch.multiple as a decorator
then the created mocks are passed into the decorated function by keyword.

```
>>> thing = object()
>>> other = object()

>>> @patch.multiple('__main__', thing=DEFAULT, other=DEFAULT)
... def test_function(thing, other):
...     assert isinstance(thing, MagicMock)
...     assert isinstance(other, MagicMock)
...
>>> test_function()
```

patch.multiple can be nested with other patch decorators, but put arguments
passed by keyword *after* any of the standard arguments created by patch:

```
>>> @patch('sys.exit')
... @patch.multiple('__main__', thing=DEFAULT, other=DEFAULT)
... def test_function(mock_exit, other, thing):
...     assert 'other' in repr(other)
...     assert 'thing' in repr(thing)
...     assert 'exit' in repr(mock_exit)
...
>>> test_function()
```

If patch.multiple is used as a context manager, the value returned by the
context manger is a dictionary where created mocks are keyed by name:

```
>>> with patch.multiple('__main__', thing=DEFAULT, other=DEFAULT) as values:
...     assert 'other' in repr(values['other'])
...     assert 'thing' in repr(values['thing'])
...     assert values['thing'] is thing
...     assert values['other'] is other
...
```

## patch methods: start and stop¶

All the patchers have start and stop methods. These make it simpler to do
patching in setUp methods or where you want to do multiple patches without
nesting decorators or with statements.

To use them call patch, patch.object or patch.dict as normal and keep a
reference to the returned patcher object. You can then call start to put
the patch in place and stop to undo it.

If you are using patch to create a mock for you then it will be returned by
the call to patcher.start.

```
>>> patcher = patch('package.module.ClassName')
>>> from package import module
>>> original = module.ClassName
>>> new_mock = patcher.start()
>>> assert module.ClassName is not original
>>> assert module.ClassName is new_mock
>>> patcher.stop()
>>> assert module.ClassName is original
>>> assert module.ClassName is not new_mock
```

A typical use case for this might be for doing multiple patches in the setUp
method of a TestCase:

```
>>> class MyTest(TestCase):
...     def setUp(self):
...         self.patcher1 = patch('package.module.Class1')
...         self.patcher2 = patch('package.module.Class2')
...         self.MockClass1 = self.patcher1.start()
...         self.MockClass2 = self.patcher2.start()
...
...     def tearDown(self):
...         self.patcher1.stop()
...         self.patcher2.stop()
...
...     def test_something(self):
...         assert package.module.Class1 is self.MockClass1
...         assert package.module.Class2 is self.MockClass2
...
>>> MyTest('test_something').run()
```

Caution

If you use this technique you must ensure that the patching is “undone” by
calling stop. This can be fiddlier than you might think, because if an
exception is raised in the setUp then tearDown is not called. unittest2 cleanup functions make this
easier.

```
>>> class MyTest(TestCase):
...     def setUp(self):
...         patcher = patch('package.module.Class')
...         self.MockClass = patcher.start()
...         self.addCleanup(patcher.stop)
...
...     def test_something(self):
...         assert package.module.Class is self.MockClass
...
>>> MyTest('test_something').run()
```

As an added bonus you no longer need to keep a reference to the patcher
object.

It is also possible to stop all patches which have been started by using
patch.stopall.

patch.stopall()¶
:   Stop all active patches. Only stops patches started with start.

## TEST\_PREFIX¶

All of the patchers can be used as class decorators. When used in this way
they wrap every test method on the class. The patchers recognise methods that
start with test as being test methods. This is the same way that the
unittest.TestLoader finds test methods by default.

It is possible that you want to use a different prefix for your tests. You can
inform the patchers of the different prefix by setting patch.TEST\_PREFIX:

```
>>> patch.TEST_PREFIX = 'foo'
>>> value = 3
>>>
>>> @patch('__main__.value', 'not three')
... class Thing(object):
...     def foo_one(self):
...         print value
...     def foo_two(self):
...         print value
...
>>>
>>> Thing().foo_one()
not three
>>> Thing().foo_two()
not three
>>> value
3
```

## Nesting Patch Decorators¶

If you want to perform multiple patches then you can simply stack up the
decorators.

You can stack up multiple patch decorators using this pattern:

```
>>> @patch.object(SomeClass, 'class_method')
... @patch.object(SomeClass, 'static_method')
... def test(mock1, mock2):
...     assert SomeClass.static_method is mock1
...     assert SomeClass.class_method is mock2
...     SomeClass.static_method('foo')
...     SomeClass.class_method('bar')
...     return mock1, mock2
...
>>> mock1, mock2 = test()
>>> mock1.assert_called_once_with('foo')
>>> mock2.assert_called_once_with('bar')
```

Note that the decorators are applied from the bottom upwards. This is the
standard way that Python applies decorators. The order of the created mocks
passed into your test function matches this order.

Like all context-managers patches can be nested using contextlib’s nested
function; *every* patching will appear in the tuple after “as”:

```
>>> from contextlib import nested
>>> with nested(
...         patch('package.module.ClassName1'),
...         patch('package.module.ClassName2')
...     ) as (MockClass1, MockClass2):
...     assert package.module.ClassName1 is MockClass1
...     assert package.module.ClassName2 is MockClass2
...
```

## Where to patch¶

patch works by (temporarily) changing the object that a *name* points to with
another one. There can be many names pointing to any individual object, so
for patching to work you must ensure that you patch the name used by the system
under test.

The basic principle is that you patch where an object is *looked up*, which
is not necessarily the same place as where it is defined. A couple of
examples will help to clarify this.

Imagine we have a project that we want to test with the following structure:

```
a.py
    -> Defines SomeClass

b.py
    -> from a import SomeClass
    -> some_function instantiates SomeClass
```

Now we want to test some\_function but we want to mock out SomeClass using
patch. The problem is that when we import module b, which we will have to
do then it imports SomeClass from module a. If we use patch to mock out
a.SomeClass then it will have no effect on our test; module b already has a
reference to the *real* SomeClass and it looks like our patching had no
effect.

The key is to patch out SomeClass where it is used (or where it is looked up
). In this case some\_function will actually look up SomeClass in module b,
where we have imported it. The patching should look like:

> @patch(‘b.SomeClass’)

However, consider the alternative scenario where instead of from a import
SomeClass module b does import a and some\_function uses a.SomeClass. Both
of these import forms are common. In this case the class we want to patch is
being looked up on the a module and so we have to patch a.SomeClass instead:

> @patch(‘a.SomeClass’)

## Patching Descriptors and Proxy Objects¶

Since version 0.6.0 both patch and patch.object have been able to correctly
patch and restore descriptors: class methods, static methods and properties.
You should patch these on the *class* rather than an instance.

Since version 0.7.0 patch and patch.object work correctly with some objects
that proxy attribute access, like the django setttings object.

Note

In django import settings and from django.conf import settings
return different objects. If you are using libraries / apps that do both you
may have to patch both. Grrr...

### Table Of Contents

- Patch Decorators
  - patch
  - patch.object
  - patch.dict
  - patch.multiple
  - patch methods: start and stop
  - TEST\_PREFIX
  - Nesting Patch Decorators
  - Where to patch
  - Patching Descriptors and Proxy Objects

#### Previous topic

The Mock Class

#### Next topic

Helpers

### This Page

- Show Source

### Quick search


Enter search terms or a module, class or function name.

### Navigation

- index
- next |
- previous |
- Mock 1.0.1 documentation »

© Copyright 2007-2012, Michael Foord & the mock team.
Last updated on Nov 05, 2012.
Created using Sphinx 1.1.3.
